# TB vaccine development: monitoring international patent filings to anticipate access challenges

**DOI:** 10.3389/fpubh.2025.1726153

**Published:** 2026-01-16

**Authors:** María Florencia Pignataro, Carolinne Thays Scopel, María Lorena Bacigalupo, Mike Frick, Sergiy Kondratyuk, Gabriela Costa Chaves

**Affiliations:** 1 Instituto de Biociencias, Biotecnología y Biología Traslacional (iB3), Universidad de Buenos Aires, Departamento de Fisiología, Biología Molecular y Celular (DFBMC), Facultad de Ciencias Exactas y Naturales (FCEN), Universidad de Buenos Aires (UBA), Buenos Aires, Argentina; 2 Consejo Nacional de Investigaciones Científicas y Técnicas (CONICET), Godoy Cruz 2290 (C1425FQB) CABA, República Argentina; 3 International Treatment Preparedness Coalition Global (ITPC-Global), Wedgewood Link, Bryanston, South Africa; 4 Treatment Action Group, New York, NY, United States; 5 Intellectual Property Scientific Research Institute of the National Academy of Legal Sciences of Ukraine, Kyiv, Ukraine; 6 Global Health Centre, Graduate Institute of International and Development Studies, Geneva, Switzerland

**Keywords:** access to essential medicines and health technologies, developing countries, intellectual property, patents, tuberculosis vaccines

## Abstract

**Introduction:**

Every year, an estimated 10 million new cases of tuberculosis (TB) are diagnosed, and 1.6 million deaths occur worldwide due to this disease. The lack of new health technologies has affected the response to the global epidemic and control of this disease. The global TB vaccine pipeline has 17 candidates in clinical trials. Stakeholders are actively beginning readiness activities to prepare, introduce and deliver new TB vaccines that prove safe and effective in a timely and equitable way. This study develops a patent landscape for late-stage TB vaccine candidates. Our findings constitute a tool to anticipate global access challenges.

**Methods:**

Two vaccine candidates were selected (MTBVAC and M72/AS01E) based on their development stage and platform features. The international patent landscape for the vaccines and for a key adjuvant component (QS-21) was performed. Importantly, our methodology encompassed identification of developers in the different steps, patent search of the applications through the Patent Cooperation Treaty (PCT), content analysis of the claims and estimates of patent term. National and regional patent filings were searched, focusing the analysis on the 30 high TB burden countries.

**Results:**

Six PCT applications for MTBVAC, 22 for M72/AS01E and 21 for the formulation adjuvant QS-21 were analyzed, potentially extending the protection period and constituting a barrier to the development/production if filed and granted at the national level. National patent applications were identified in high TB-burden countries (e.g., Brazil and India), as well as in countries where the natural source of the adjuvant is mainly grown and extracted (Chile).

**Discussion:**

Our results show that there were patent filings in countries with existing manufacturing capacity, unveiling a corporate strategy to include those countries. The study anticipates the potential access challenges related to intellectual property barriers for expanding manufacturing capacity in middle-income countries to allow future TB vaccines to be available in a timely and equitably manner for those most in need.

## 1 Introduction

Tuberculosis (TB) is a poverty-related TB epidemic disease responsible for an estimated 10 million new cases and 1.6 million deaths every year. Thirty countries—the majority low- and middle-income countries (LMIC)—are classified as high TB burden for at least TB, HIV-associated TB and/or multi-drug per multidrug resistant/rifampicin-resistant TB (MDR/RR-TB) (1). In 2015, the World Health Organization (WHO) launched “The End TB Strategy” (2) following Member States’ commitment (3) to reduce deaths by 95% and incidence by 90% compared to 2015 levels. Governments reaffirmed this commitment to end TB at two United Nations (UN) General Assembly High-Level Meetings on TB in 2018 and 2023. Importantly, the political declaration of the 2023 UN High-Level Meeting included a commitment to “accelerate the research, development and roll-out of safe, effective, affordable and accessible pre- and post-exposure vaccines, preferably within the next 5 years,” which would set a deadline in 2028 (4).

Despite killing an estimated 1 billion people over the last two centuries (5), for decades there has been a gap in the development of health tools—diagnostics, vaccines and therapeutics—to respond to the global TB epidemic. Funding for TB research and development (R&D) remains neglected, at around US$ 1 billion per year globally, and has fallen short of UN goals to spend at least US$ 5 billion per year. Within TB research, vaccine R&D stands out as the area of greatest need: in 2023, combined spending on TB vaccine research by governments, philanthropies, pharmaceutical companies, and multilateral organizations amounted to only US$ 227 million, or less than 20% of the US$ 1.25 billion funding target (6).

Starting only from the mid-2000s, there was a successful effort to develop and approve a molecular diagnostic platform (GeneXpert) that is simple to use at the district level and that provides timely results (7). The only licensed vaccine for TB—BCG—was introduced in 1921 (8). Therapeutics were mainly developed in the first half of the 20th century (9), during the initial antibiotics boom, followed by the antibiotics development void from the 1980s and 1990s (10) and the rise of multidrug-resistant TB (MDR-TB). The first new medicines for MDR-TB—bedaquiline and delamanid—were only first approved, respectively, in 2012 (US Food and Drug Administration—FDA) and 2014 (European Medicines Agency—EMA) (11). At the time of their approval, they were the first new compounds from novel classes developed for TB in over 40 years.

Progress in TB vaccine development has starkly contrasted with the rapid advancements seen during the Covid-19 pandemic. However, political momentum to develop new TB vaccines is starting to be built with the creation of a special initiative of the WHO Director-General called the TB Vaccine Accelerator Council “to catalyze high-level commitment, engagement, and alignment among funders, global agencies, governments, and communities, to identify and overcome the major barriers to tuberculosis vaccine development” (12).

Currently, the global TB vaccine pipeline has 17 different candidates in clinical trials, six of which have reached efficacy trials (phase II and III). To date, no TB vaccine candidates have been approved for emergency use authorization (13, 14). From the six candidates in phase III clinical trials, three are live attenuated vaccine candidates (MTBVAC, BCG travel vaccine and VPM1002), two are protein/adjuvant vaccines (M72/AS01^E^ and GamTBvac) and one is an inactivated vaccine (Immuvac or MIP) (15). Among the most anticipated studies are a phase III clinical trial of M72/AS01^E^ (NCT06062238; sponsored by the Gates Medical Research Institute and funded by the Gates Foundation and Wellcome) and a phase IIb trial of MTBVAC (NCT06272812; sponsored by Biofabri and IAVI with funding from the Gates Foundation, Open Philanthropy, and German government). Both trials are testing whether the vaccines prevent TB disease among adolescents and adults.

In this context, with multiple candidates in late-stage trials, the WHO and several Civil Society Organizations (CSOs) are actively beginning readiness activities to prepare, introduce and deliver new TB vaccines that prove safe and effective in a timely and equitable way. Some of the main recommended actions are to strengthen the local capacities of supply and manufacturing (involving global procurement agencies), enhance policy and regulatory strategies, and ensure sustainable financing along with political engagement (16). Some CSOs are particularly concerned with access in middle income countries (MIC), including some high TB burden countries, that are not eligible by Gavi (the Vaccine Alliance) for the Vaccine Investment Strategy (VIS), calling to actions toward developing investment strategies in vaccine delivery and promoting mechanisms for pool procurement (17). Therefore, beyond scientific and technical readiness, sustained political commitment and international cooperation will be essential to ensure equitable vaccine access (18).

There have been access challenges for new technologies due to the monopoly situation and high prices. The development of technologies such as GeneXpert (7) and bedaquiline (19) relied mostly on public funding and yet their approval was not followed by affordable prices. Advocates and representatives of TB-affected communities have campaigned (20) for more transparency on the cost-of-goods and for a price reduction of GeneXpert. Bedaquiline’s technology holder (Johnson & Johnson) decided not to enforce secondary patents in 134 LMIC (21) after campaigning and pressure from CSOs (22, 23) all over the world and from a global health institution (24). The CSOs efforts to challenge patent barriers in many countries, as India, Brazil, Thailand, Ukraine, Kazakhstan, Kyrgyzstan, Belarus, Moldova and Vietnam (23, 25) contributed to put the drug in public domain and unlock generic competition to influence price decrease, as the non-enforcement of patents did not mean automatic withdrawal of patents.

In the pharmaceutical sector, the filing of multiple patent applications related to the same active pharmaceutical ingredient (API) or components of a technology, along with other practices, aims at extending the monopoly over technologies and is known as evergreening (26). While in the scope of ‘life cycle management’ of key technologies for companies, the multiple patenting can also be a barrier to access to health technologies by creating or extending their monopoly situation, with effects on price and in the freedom to operate assessment by procurers and manufacturers.

The World Trade Organization Trade-Related Aspects of Intellectual Property Rights (WTO-TRIPS) Agreement entered into force in 1995 and established minimum standards for intellectual property rights (IPR) in which Member States would have to comply. This included the adoption of patent protection for all technological fields, including for health technologies. Since then, countries, especially LMIC, have been faced with the high price of life-saving technologies, compromising the fulfillment of access policies. Over the past decade, high priced medicines because of patent monopoly have also affected access in high income countries (HIC) (27, 28). The WTO Doha Declaration on the TRIPS Agreement and Public Health states that countries should take measures to protect public health, which encompasses the adoption of the so-called TRIPS flexibilities (29), such as research exemption, a public health approach to patent examination, patent oppositions, compulsory licensing, among others.

In November 2025, one of the outcomes of WHO TB Vaccines Accelerator Finance & Access Working Group is an analysis of the anticipated barriers, bottlenecks, and market dynamics that could impact timely, equitable, and sustainable access to novel TB vaccines. This report mentions that novel TB vaccines should be positioned as public goods and that long-term sustainable access will rely on competition, acknowledging that current pipeline indicates monopoly and oligopolistic dynamics when the first products enter the market and presence of only one global supplier that produces the adjuvant for one of the advanced candidates. As a solution report proposes that “advocacy with relevant supply stakeholders should aim to secure manufacturing capacity to produce at least one vaccine in each high-burden region (i.e., Africa, Asia and Latin America)” assuming voluntary technology transfer. Nevertheless, it does not explicitly mention how to address the monopoly in case of unwillingness of originators to share technology unduly limiting access and supply security, such as addressing intellectual property (IP) barriers through the use of TRIPS flexibilities (18).

In this context, the analysis of the patent landscape of vaccine candidates is an essential activity in the scope of readiness to prepare the introduction and delivering of new TB vaccines. This analysis is not only a way to understand the scope of protection that companies and institutions are pursuing through the development process but mostly, an approach to anticipate access and development challenges related to the components of the vaccine (either isolated or altogether) or its production process. A patent landscape can be considered one step to support a Freedom to Operate (FtO) assessment by providing an analysis on the scope of protection aimed on a specific technology and identifying in which jurisdiction there are patent filings (30). Strategies to pave the way for the strengthening of manufacturing capacity should take into account the patent landscape of these technologies (31).

This study aims to develop and analyze the patent landscape of two promising late-stage TB vaccine candidates, MTBVAC and M72/AS01^E^, to identify trends in patent protection and assess their potential impact on vaccine accessibility in LMIC with a high TB burden related to development, production, supply and affordability.

## 2 Methodology

The methodology involved a four-step approach comprising the selection of technologies and their description; patent search from selected applicants; content analysis of the claims and identification of national and regional filings and status.

A FtO assessment is defined as “the ability to proceed with the research, development and/or commercial production, marketing or use of a new product or process with a minimal risk of infringing the unlicensed IP (intellectual property) rights or TP (tangible property) rights of third parties” (32). Therefore, we assume the present analysis contributes to a FtO assessment with regards to patenting and filings in different jurisdictions, but it does not cover the entire scope of IP and TP rights nor provide guidance on specific patents at the national level with claims potentially blocking the technology.

### 2.1 Selection of health technologies

These two candidates were selected because they are undergoing late-stage efficacy trials (phase III) and of different platforms: a mycobacterial live-attenuated vaccine (MTBVAC) and a protein/adjuvant subunit vaccine (M72/AS01^E^). A difference regarding these candidates is the presence of an adjuvant in the formulation. Adjuvants are essential components for some types of vaccines, almost as important as the antigen itself (33). While MTBVAC has no adjuvant added, M72/AS01^E^ has an adjuvant (AS01^E^) at the center of many discussions regarding its supply and access (34). The adjuvant AS01^E^ consists of two immune enhancers: 3D-MPL (3’-O-desacyl-4′-monophosphoryl lipid A) and QS-21 (*Quillaja saponaria* Molina, fraction 21), in a liposome suspension.

### 2.2 Building of the patent landscape

The patent landscape for the selected vaccine candidates was built from a search in the commercial CAS Patent Explorer database (35), considering patent applications filed through the Patent Cooperation Treaty (PCT) system, publicly available up to March 2025. To narrow the patent search, specific applicants and/or assignees were selected based on their involvement in the development of each technology.

Complementary patent search was performed for QS-21, filtering as applicants and/or assignees the companies identified in the literature as being involved in the R&D or commercialization of saponins and their potential application as adjuvants. The search considered the patent applications filed through the PCT system, publicly available up to March 2025.

After using different filters, an inclusion and exclusion criteria was applied considering the components of each specific vaccine.

The searches were undertaken as following:

- M72/AS01^E^ landscape: search was conducted using “GlaxoSmithKline” and “Smithkline Beecham Biologicals” filters as applicants [SmithKline Beecham merged with Glaxo Wellcome in 2000 to become GlaxoSmithKline (GSK)].
- MTBVAC landscape: search was conducted using “MTBVAC” and “tuberculosis AND zaragoza” keywords and “University of Zaragoza” and “Biofabri” filters as applicants.
- QS-21 landscape: search was conducted using “Cambridge Corporation,” “Aquila Pharmaceuticals,” “Antigenics,” “Agenus,” “Phyton Biotech,” “Q-VANT,” “Desert King,” “Croda International,” “Botanical Solutions” and “Plant Bioscience” filters as applicants.

The patent applications were classified based on the general content of the claims of the PCT applications, adapting the classification for small molecules (36). The scope and content of the claims of the national filings were not analyzed.

To demonstrate the evergreening approach for each technology, patent applications were organized according to an estimate of 20 years patent term, starting from the international filing date, in case they are filed and granted at the national level.

### 2.3 Analysis of national and regional filings

National and regional patent filings related to the previously included PCT applications were searched in CAS Patent Explorer database and in WIPO Patent Scope and analyzed (35, 37). For a few cases, the status and national numbers were adjusted according to inputs from national search (India and Indonesia). The national and regional search considered all countries available in the mentioned databases (Supplementary Tables S1, S2) while in the main text (Tables 1, 2) focused on the 30 high TB burden countries according to the WHO 2024 report (1). Chile and Mexico were also included in the patent search related to M72/AS01^E^ vaccine candidate because the company responsible for supplying QS-21 (Desert King), from tree extraction, is based in those countries. Applications filed in regional patent offices that comprised some of these 30 high TB burden countries were also included: African Regional Intellectual Property Organization (ARIPO) and African Intellectual Property Organization (OAPI).

**Table 1:** National or regional patent applications on MTBVAC vaccine candidate in the 30 high TB burden countries.

International PCT applications	National patent filings	Valid patents (granted, not expired)
WO/2003/012075	-	-
WO/2007/110462	Brazil (BRPI0709106) China (CN101405386) India (IN268173)	Brazil (BRPI0709106) China (CN101405386) India (IN268173)
WO/2015/144960	-	-
WO/2018/006939	-	-
WO/2019/158779	Brazil (BR112020016704) China (CN116751703, CN112449604) India (IN202017038876, IN202318031011)	China (CN112449604)
WO/2021/058831	China (CN114845732) India (IN202217022701)	China (CN114845732)

Source: the authors, based on the patent status provided at WIPO Patent Scope and CAS Patent Explorer.

**Table 2:** National or regional patent applications on M72/AS01^E^ vaccine candidate in the 30 high TB burden countries (in *italics*), Chile and Mexico filed by GSK.

International PCT applications	National patent filings	Valid patents (granted, not expired)
WO/1994/000153	ARIPO* (AP408) *China (CN1122530, CN1086142)* *South Africa (ZA199304504)* —- Mexico (MX9303773)	-
WO/1996/033739	ARIPO* (AP771A) *Brazil (BRPI9608199)* *China (CN1182370,* *CN1480214, CN1515245)* *Indonesia (IDP000005617)* *India (IN2467/DEL/2007)* OAPI* (OA10629) *Thailand (TH27045)* *Vietnam (VN1068)* —- Mexico (MX9708226)	-
WO/2003/028760	-	-
WO/2006/117240	*Brazil (BRPI0611347, BRPI0622304)* *China (CN101273055, CN102617739, CN106390108, CN105903008)* *Indonesia (ID201404894, ID481049)* *India (IN296468)* *Philippines (PH12013502449,* *PH12007502365)* *Vietnam (VN10015346, VN20476)* *South Africa (ZA200709209)* —- Mexico (MX297551, MX324839, MX326059)	*India (IN296468)* *Vietnam (VN10015346)* *South Africa (ZA200709209)* — Mexico (MX324839, MX326059)
WO/2007/068907	*Brazil (BRPI0619795)* *China (CN103861100, CN103405764, CN102631670)* *India (IN2608KOLNP2008)* *Indonesia (IDP000031710)* *Philippines (PH12008501400)* *Vietnam (VN10012047)* —- Mexico (MX292604)	*Brazil (BRPI0619795)* *Philippines (PH12008501400)* *Vietnam (VN10012047)* — Mexico (MX292604)
WO/2010/142685	*Brazil (BRPI1012890)* *China (CN104367997, CN102458457)* *India (IN279396)* *Thailand (TH125812)* *Vietnam (VN30393)* *South Africa (ZA201108507)* — Chile (CL201103113) Mexico (MX325138)	*Brazil (BRPI1012890)* *China (CN104367997, CN102458457)* *India (IN279396)* *South Africa (ZA201108507*) —- Mexico (MX325138)
WO/2011/144645	*Brazil (BR112012028930)* *China (CN102933606)* *India (*IN322114*)*	*Brazil (BR112012028930)* *China (CN102933606)* *India (IN322114)*
WO/2012/080370	*Brazil (BR112013014598)* *China (CN103260642, CN106822882, CN106822883, CN103249431)* *India (IN342832)* *Indonesia (IDP000040568, ID201402335)* *Philippines (PH12013501204)* *Thailand (TH143305)* *Vietnam (VN36110, VN35845)* *South Africa (ZA201304014, ZA201304015)* *—* Mexico *(MX344280)*	*China (CN103249431, CN103260642)* *South Africa (ZA201304014)* — Mexico (MX344280)
WO/2012/080369	*Brazil (BR112013014599)* *China (CN103249431, CN103260642, CN106822882, CN106822883)* *India (IN1650KOLNP2013)* *Indonesia (IDP000040568, ID201402335)* *Philippines (PH12013501203)* *Thailand (TH143304)* *Vietnam (VN35845, VN36110)* *South Africa (ZA201304014, ZA201304015)* —- Mexico (MX344280, MX344706)	*China (CN103249431, CN103260642)* *South Africa (ZA201304014, ZA201304015)* —- Mexico (MX344280, MX344706)
WO/2015/150567	*Brazil (BR112016022787, BR112016022463)* *China (CN106456738, CN106456739)* *India (IN201617030399, IN201617031463)* *South Africa (ZA201605955)* — Mexico (MX2022013912, MX2016012932, MX401336)	*China (CN106456738)* *South Africa (ZA201605955)* — Mexico (MX401336)
WO/2017/102737	-	-
WO/2018/104313	*Brazil (BR112019011286)* *China (CN110035770)* *India (IN544265)* — Mexico (MX2019006728)	*China (CN110035770)*
WO/2018/114892	*China (CN110290806)*	-
WO/2018/219521	*Brazil (BR112019025193)* *China (CN111032080)* *India (IN201917053739)* — Mexico (MX2019014319, MX2023012979, MX2023012980, MX2023013020)	*-*
WO/2018/206776	*China (CN110891595)* *India (201917049171)* *South Africa (ZA201907459)*	*South Africa (ZA201907459)*
WO/2019/106191	*Brazil (BR112020010635)* *China (CN111372604)* *India (IN202017023446*) — Chile (CL202001439) Mexico (MX2020005480)	-
WO/2019/106192	*Brazil (BR112020010790)* *China (CN111670044)* *India (IN202017023959)* — Chile (CL202001440) Mexico (MX413557)	Mexico (MX413557)
WO/2021/224205	*China (CN115485057)* *Brazil (BR112022020660)* *India (IN202217059500)* — Mexico (MX2022013855)	-
WO/2022/122830	*Brazil (BR112023010982)* *India (202317037525)* — Chile (CL202301630) Mexico (MX2023006769)	-
WO/2023/066885	*India (IN202417036649)* *–* Chile (CL202401193)	-
WO/2023/242187	*China (CN119403935)* *India (IN202417095096)* *—* Chile (CL202403833)	-

*Regional patent offices comprising African countries. Source: the authors, based on patent status analysis from WIPO Patent Scope, CAS Patent Explorer and national searches (India, Indonesia).

## 3 Results

### 3.1 MTBVAC vaccine candidate

MTBVAC is a live rationally attenuated vaccine candidate derived from the *Mycobacterium tuberculosis* isolated MT103 (lineage 4, Euro-American), one of the most widespread lineages of *M. tuberculosis*. There are two independent stable deletion mutations in the virulence genes: phoP (Rv0757) and fadD26 (Rv2930). The first gene is related to the production of immunomodulatory cell-wall lipids and ESAT-6 secretion; and the second is related to the biosynthesis of the virulence surface lipid phthiocerol dimycocerosate (PDIM).

MTBVAC was developed by the University of Zaragoza (in Spain) with Pasteur Institute (in France). The biopharmaceutical company Biofabri, which is part of the transnational group Zendal, oversees the clinical and industrial development through several partnerships. There is a globally-focused partnership between Biofabri and the International AIDS Vaccine Initiative (IAVI), an India-focused partnership between Biofabri and Bharat Biotech International Limited (BBIL), and a European Union-funded partnership in which Biofabri is studying MTBVAC as an infant vaccine (38).

**Figure 1:**
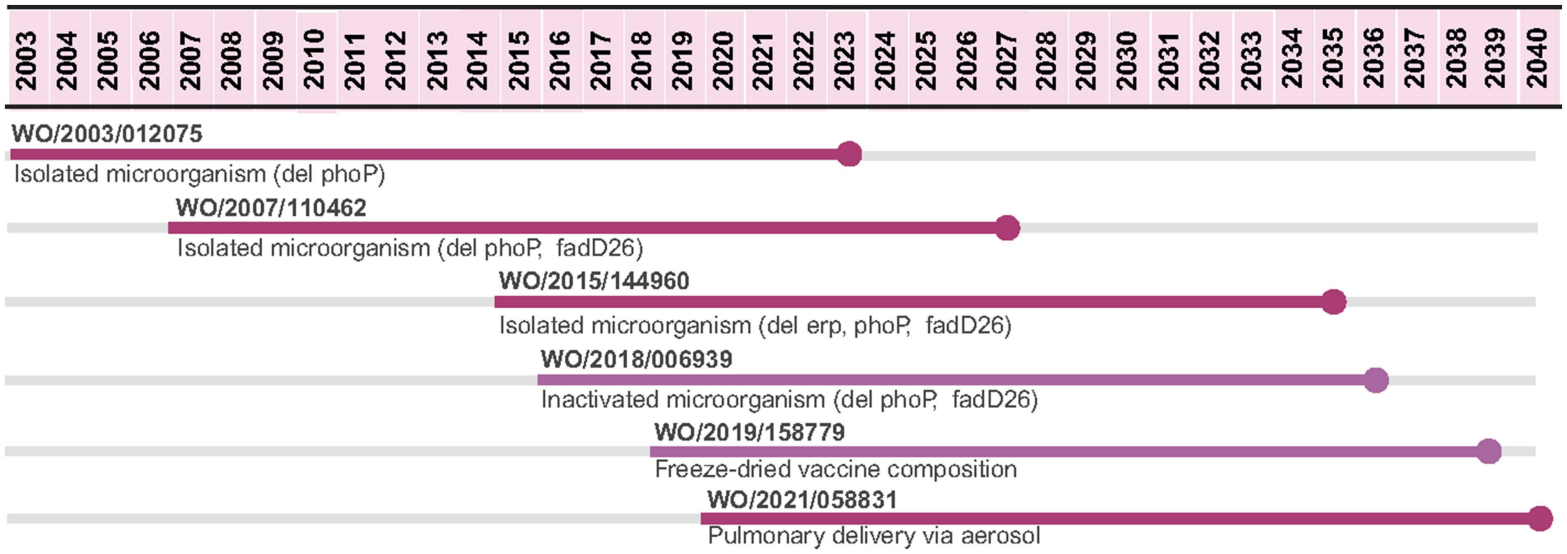
PCT applications related to MTBVAC vaccine candidate. Patent applications were filed by University of Zaragoza (dark purple) and by University of Zaragoza and Biofabri (light purple). Source: the authors, based on patent search and content analysis of the claims. Created in Biorender (https://BioRender.com).

Regarding international patent filing, six PCT applications filed by the University of Zaragoza and Biofabri have been identified since 2003 (Figure 1), involving the patent protection of the microorganism (four patent applications) or pharmaceutical compositions (two patent applications).

The first PCT application covers the isolated microorganism with phoP deletion with reduced virulence; meanwhile the following PCT application covers the isolated microorganism with an additional deletion (phoP and fadD26) and the related vaccine. The double mutant microorganism is the API of the MTBVAC vaccine. The third PCT application covers a triple mutant microorganism (phoP, fadD26 and erp) aimed to provide a more attenuated phenotype able to vaccinate patients at risk of immunosuppression. WO/2018/006939 discloses the inactivated version of MTBVAC, referred to as MTBVAC+ (39, 40). The last two PCT applications filed are related to pharmaceutical compositions: freeze-dried and pulmonary delivery via aerosol (the pharmaceutical composition used in the clinical trials is the freeze-dried formulation) (41).

**Figure 2:**
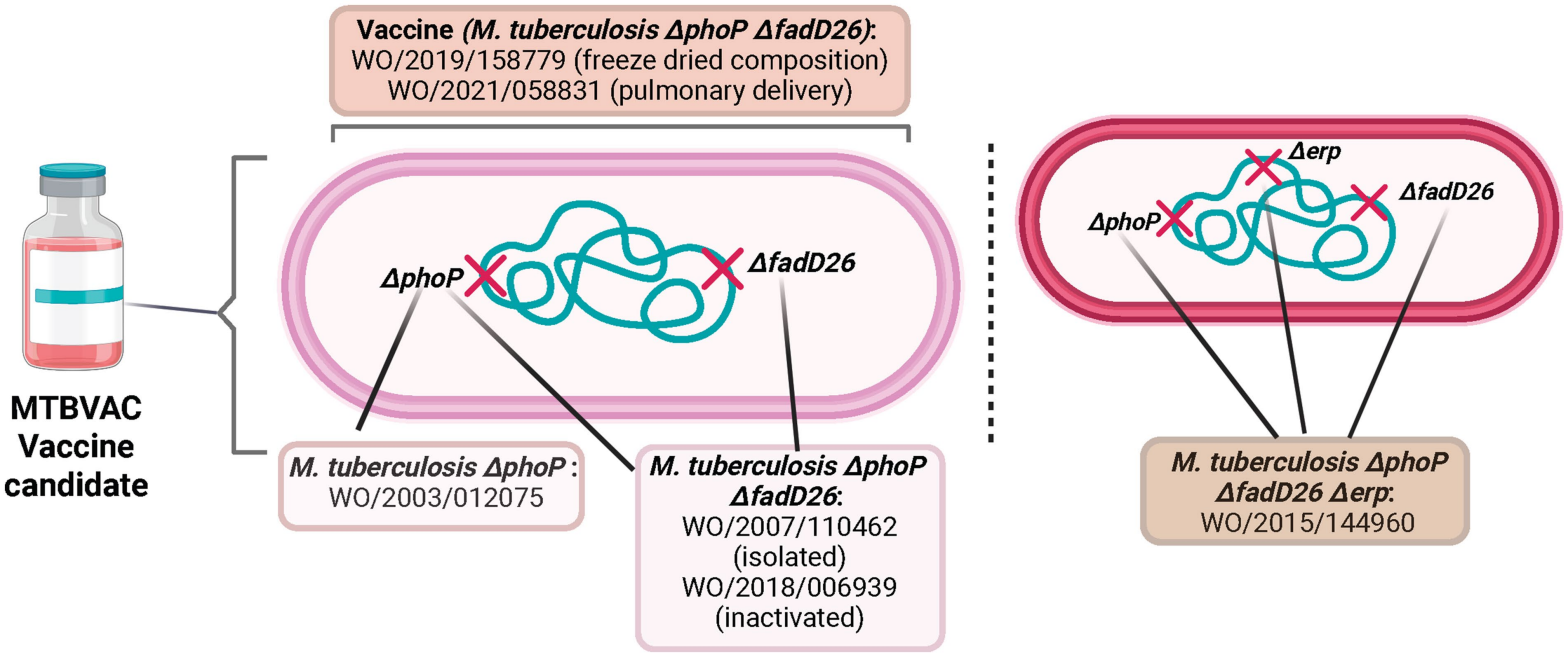
PCT applications covering specific components of the MTBVAC vaccine candidate. Source: the authors, based on patent search and content analysis of the claims. Created in Biorender (https://BioRender.com).

Considering a 20-year patent term, if the last PCT application is filed and granted in a country, the patent is expected to expire in 2040, 17 years after the expiring date of the first PCT application (Figure 1). Based on the content analysis of the claims, Figure 2 describes how different components of MTBVAC are covered by different PCT applications.

**Figure 3:**
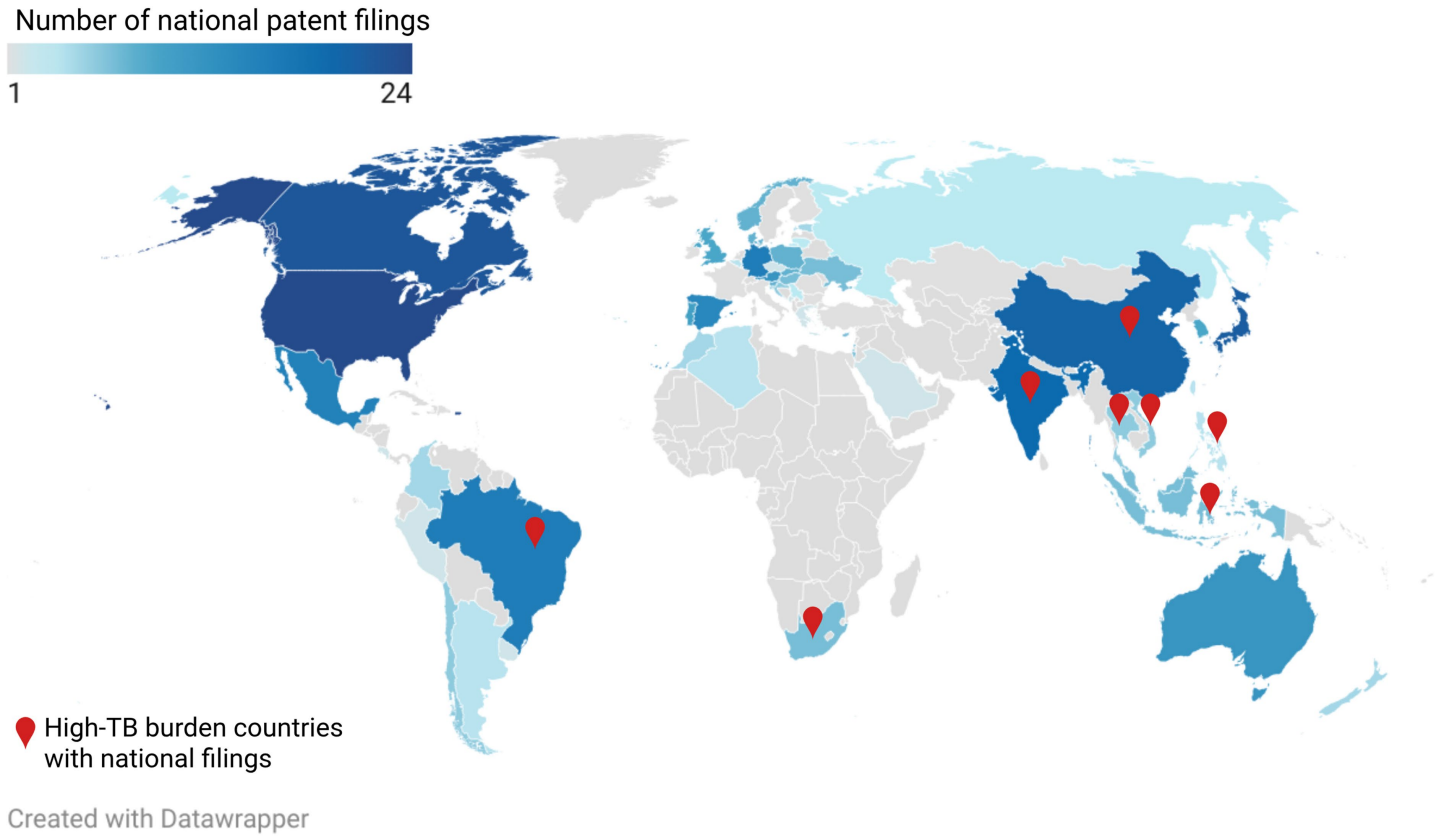
Global distribution of national patent filings related to MTBVAC and M72/AS01^E^ vaccine candidates. The map shows the number of patent filings per country (color gradient) and highlights countries with patent filings that are included in the list of High-TB burden countries (red symbol). Whenever multiple divisional applications were filed in the same country or regional patent office, only one was counted in the analysis, representing the national phase entry of a single PCT application. Patent filings submitted to regional patent offices were not included in the figure. The not included regional patent offices are: African Regional Intellectual Property Organization (ARIPO, two filings); African Intellectual Property Organization (OAPI, one filing); Gulf Cooperation Council Patent Office (GCCPO, one filing); Eurasian Patent Office (EAPO, 7 filings); European Patent Office (EPO, 25 filings). Source: the authors, based on the patent search produced for this article. Created with Datawrapper and Biorender (https://BioRender.com).

In relation to the national or regional patent filings (Table 1; Supplementary Table S1; Figure 3), applications were found in both HIC, which are not high TB burden (such as United States of America-USA, Canada, Austria, Spain, Portugal, Australia, Japan, Russia and the European patent office- EPO) and in three of the 30 high TB burden countries (Brazil, China and India).

Granted patents related to the API were identified in three high TB burden countries: Brazil, China and India. One granted patent covers the pulmonary delivery via aerosol formulation in China.

### 3.2 M72/AS01^E^ vaccine candidate

M72/AS01^E^ is a subunit vaccine candidate that contains the M72 recombinant fusion protein derived from two immunogenic *M. tuberculosis* antigens: Mtb32A and Mtb39A, combined with the AS01^E^ adjuvant system, which is also a component of the malaria vaccine (Mosquirix, RTS, S/AS01) and recombinant Herpes Zoster vaccine (Shingrix), both commercialized by GSK.

The AS01^E^ is the lower dose version of the same adjuvant used in Shingrix vaccine (AS01^B^) supposedly optimized for tolerability (42). Importantly, both components of the AS01 system rely on a good safety record profile (43). 3D-MPL (44) is a non-toxic derivative of bacterial lipopolysaccharide (LPS, commonly known as endotoxins). Particularly, 3D-MPL is a purified, non-toxic endotoxin derivative obtained from *S. minnesota*, manufactured by Corixa Corporation (GSK Vaccines). MPL is a well characterized substance (45). The biological properties of MPL are attributed to its immunostimulatory effects on the innate immune system (via activation of the toll-like receptor 4) and the direct activation of antigen presenting cells (APC) resulting in enhanced phagocytosis and microbicidal activities because of the production of IL-12, TNF-*α*, GM-CSF, and IFN-*γ* (46, 47).

QS-21 contains a mixture of structurally related saponins (triterpene glycosides) obtained by chromatographic purification of an aqueous extract of the bark of the so-called Soapbark tree, *Quillaja saponaria* Molina (48, 49). Aqueous extracts from the Soapbark were extensively used in animal vaccines (under the name QuilA). QuilA is unsuitable for human use due to its toxicity, differential purifications of the crude extract led to four fractions, QS-21 being the least toxic in animal models (48). QS-21 promotes both humoral (Th2) and cell-mediated (Th1) immunity when added to vaccine formulations through action on APCs and T cells (50).

M72/AS01^E^ vaccine was initially developed by GSK as an investigational candidate and evolved to clinical development (phase IIb study) in a partnership between Aeras and GSK (51).

**Figure 4:**
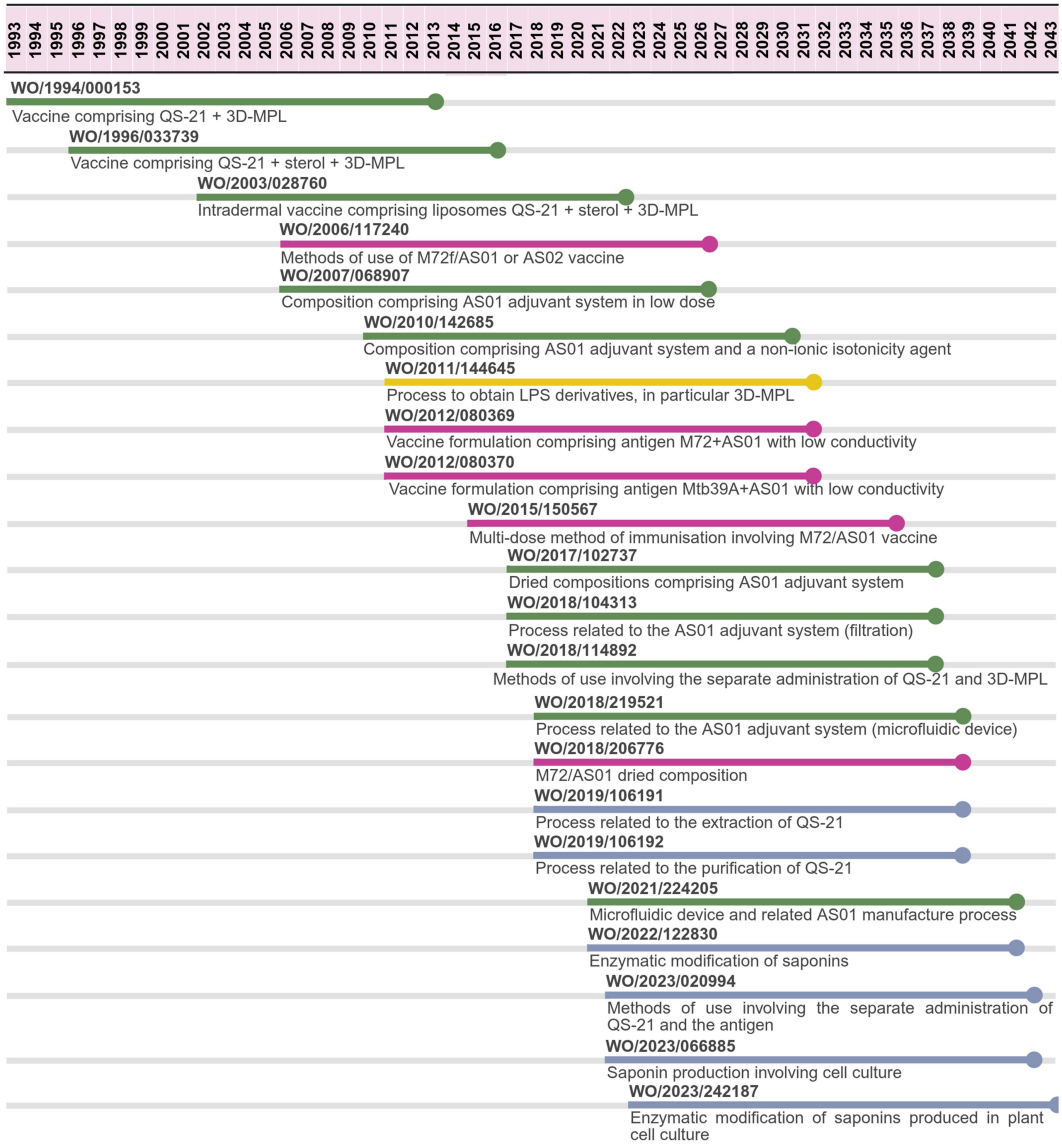
PCT applications related to M72/AS01^E^ vaccine candidate filed by GSK. Patent applications may focus on the adjuvant system (green)—or its individual components 3D-MPL (yellow) or QS-21 (blue)-, or on the tuberculosis vaccine (purple). Source: the authors, based on patent search and content analysis of the claims. Created in Biorender (https://BioRender.com).

In relation to international patent filings (Figure 4), 22 PCT applications filed by GSK and related divisions have been identified since 1993. From all the PCT applications, only five are related to a TB vaccine, meanwhile 17 are related to the adjuvant: 11 to the adjuvant system comprising 3D-MPL + QS-21 (covering the process, formulations or methods of use); five to processes related to the adjuvant QS-21 and one to the adjuvant 3D-MPL obtention.

The first two PCT applications identified refer to broad compositions comprising an adjuvant system of QS-21 and 3D-MPL (covering both AS01 and AS02), meanwhile the third refers to the same adjuvants formulated in liposomes (AS01) for an intradermal vaccine. WO/2006/117240 is the first one referring to a TB vaccine, specifically to methods to prevent the reactivation of TB by administering the composition comprising M72f antigen and QS-21 and 3D-MPL (AS01 or AS02).

The following two PCT focus on the adjuvant system AS01: WO/2007/068907 refers to the dose of the adjuvants and WO/2010/142685 refers to the presence of a non-ionic isotonicity agent. WO/2011/144645 covers a process to obtain LPS derivatives (3D-MPL).

From 2019, five out of seven PCT applications refer to processes related to QS-21, including extraction, purification and enzymatic modification (WO/2019/106191, WO/2019/106192, WO/2022/122830, WO/2023/066885, WO/2023/242187) with the last two specifying the inclusion of cell culture methods.

Three subsequent PCT applications are related to the TB vaccine M72/AS01^E^. The first two cover compositions comprising M72 or Mtb39A antigen with certain composition features, such as the ability to conduct electricity, given by its components; meanwhile WO/2015/150567 claims a multi-dose method of immunization. The following three (WO/2017/102737, WO/2018/104313, WO/2018/114892) cover AS01 formulation, production or methods of immunization comprising the separate administration of each adjuvant (not co-formulated). WO/2018/219521 refers to a dried composition of M72/AS01 vaccine.

WO/2021/224205 refers to a microfluidic device and process related to AS01 process, meanwhile WO/2023/020994 covers methods of use involving a first administration of the saponin formulation prior to the antigen.

**Figure 5:**
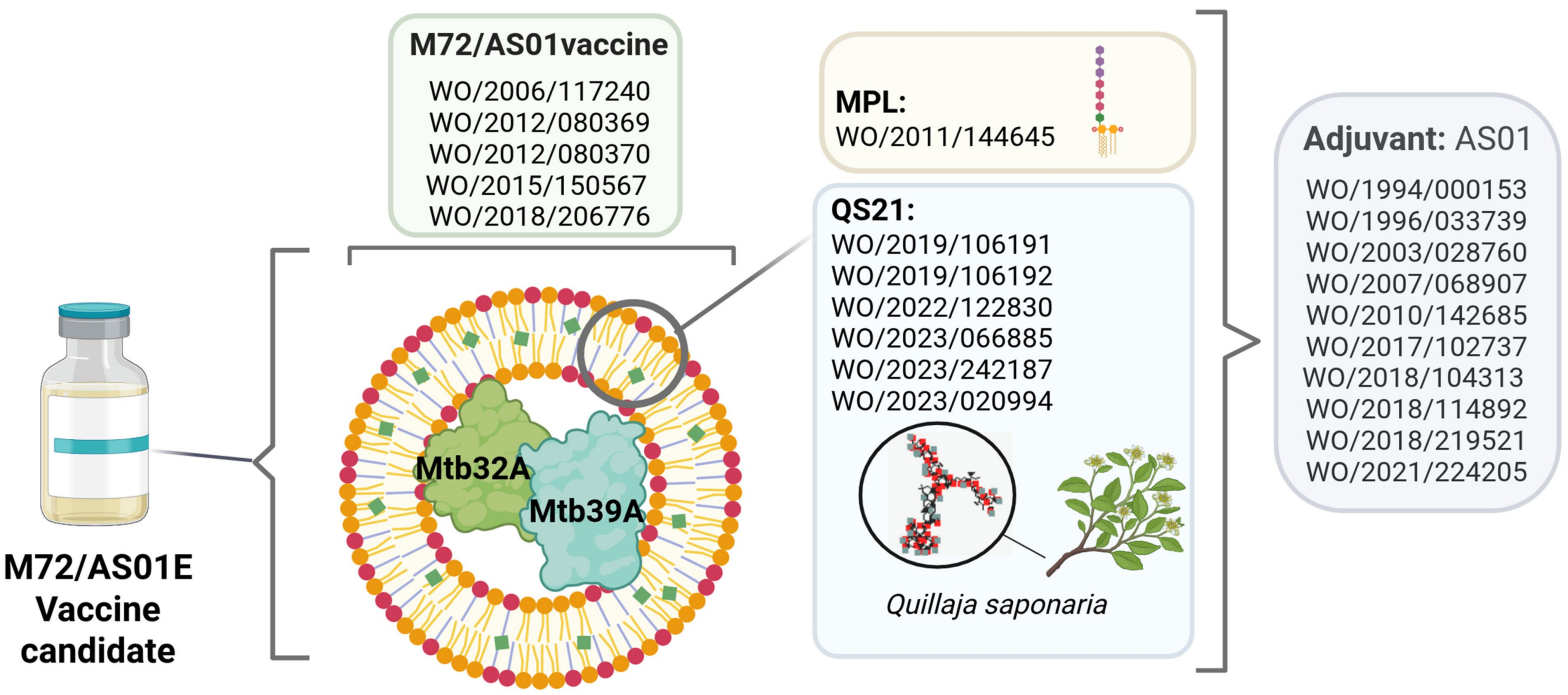
PCT applications covering specific components of the M72/AS01^E^ vaccine candidate filed by GSK. Source: the authors, based on patent search and content analysis of the claims. Created in Biorender (https://BioRender.com).

Considering a 20-year patent term, if the last PCT application is filed and granted in a country, the patent is expected to expire in 2043, 30 years after the expiring date of the first PCT application (Figure 4). Based on the content analysis of the claims, Figure 5 describes specific components of the M72/AS01^E^ vaccine candidate covered by the different PCT applications.

In relation to regional or national patent filings (Table 2; Supplementary Table S2; Figure 3), most of the patent applications from the year 1996 to 2012 are expired, withdrawn or rejected in an extended list of countries (complete list could be found in Supplementary Table S2).

The vast majority of patent applications were identified in both HIC (such as United States, Canada, Austria, Spain, Portugal, Australia, Japan, Russia and the EPO) and in some MIC with high TB burden (such as Brazil, China, India, Indonesia, Philippines, Thailand, Vietnam and South Africa) (1), showing that the applicant pursued the protection at the national level particularly in countries where the TB rates are high. There are patents granted in Brazil, China, India, Indonesia, Philippines, South Africa and Vietnam.

Importantly, the national applications related to the PCT application WO/2012/080370 (covering a M72/AS01^E^ composition) were mostly granted, including China, South Africa and Vietnam (Supplementary Table S2). Moreover, national applications related to WO/2012/080369 and WO/2015/150567—covering M72/AS01^E^ composition and methods of use, respectively—were also granted in China and South Africa while they were rejected in India (Supplementary Table S2).

Most of the patent applications related to the adjuvant systems (filed from 2017 and onwards) seem to be filed also in some high TB burden countries (for example India and China) and while most of them are still pending, a few were already granted. The newer PCT applications related to the saponin manufacture were filed in several countries, including HIC such as United States, Canada, Japan, EPO, as well as high TB burden countries (China, Brazil, India), and in Chile, where the main natural source of QS-21 grows.

**Figure 6:**
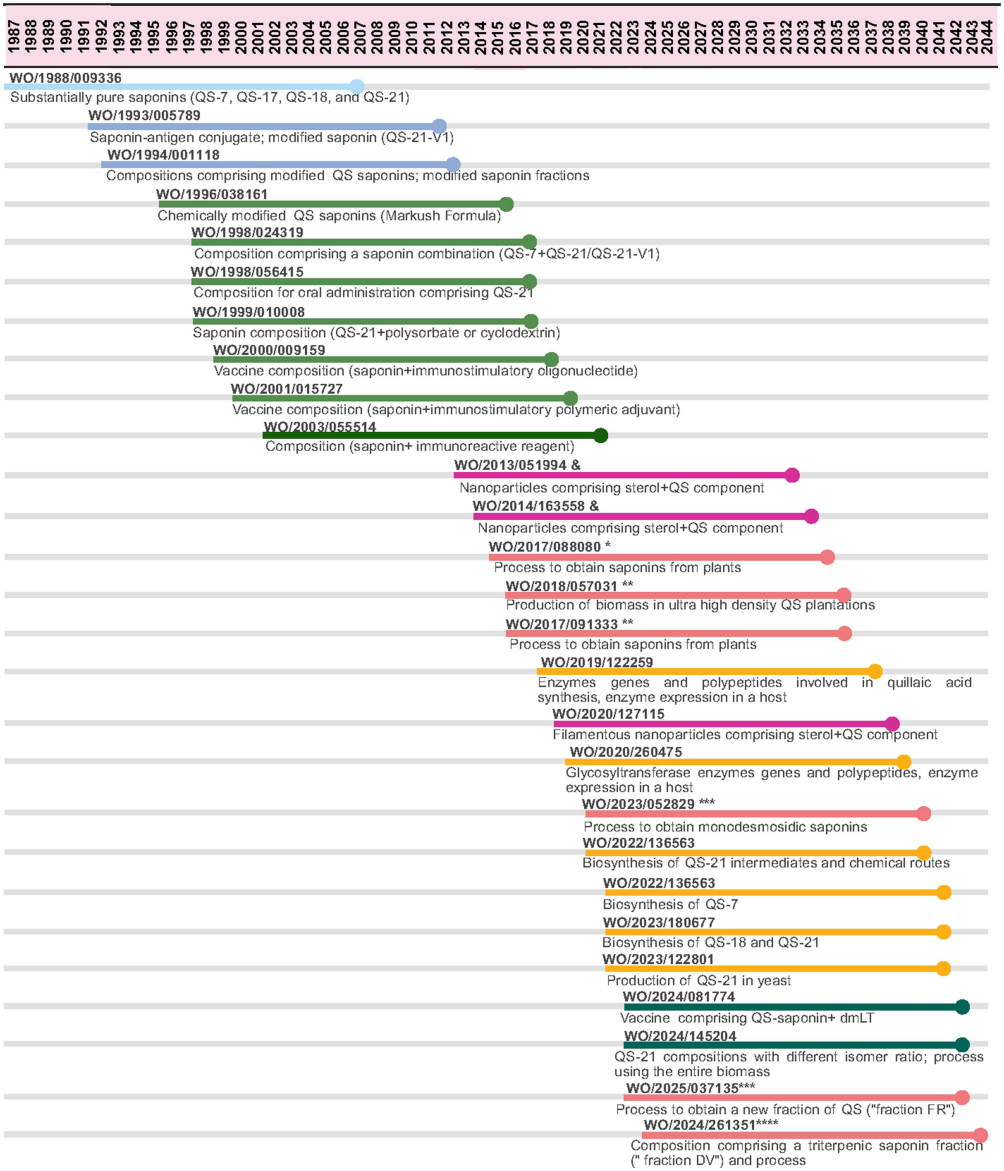
PCT applications by different applicants related to saponins from Quillaja Saponaria. Patent applications were filed by Cambridge Bioscience Corporation (light blue), Cambridge Biotech Corporation (blue), Aquila Biopharmaceuticals Inc. (light green), Antigenics Inc. (dark green), Desert King (pink; *International Application Applicant: Natural Response S. A.; Chilean Application Applicant: Desert King Chile; **Co-Applicants: Natural Response S. A.; Desert King International Llc; ***Applicant: Desert King Chile; ****Co-Applicants: Vaccine Formulation Institute Ch Ltd.; Desert King International Llc), Q-Vant Biosciences (green), Croda International LLC (purple, &International Application Applicant: Moreinx Ab; Europe, Canada And Us Applicant: Croda International LLC), Plant Bioscience Limited (yellow). Source: the authors, based on patent search and content analysis of the claims. Created in Biorender (https://BioRender.com).

Figure 6 provides an overview of PCT applications by different applicants and/or assignees related to saponins from *Quillaja saponaria*, including QS-21, and different approaches of protection by different institutions over time. Initial applications focused on the saponins as compounds or their chemical modifications, others are related to pharmaceutical compositions, such as vaccines, up to process type of claims, including their extraction from plants. The applicants and/or assignees of QS-21 PCT applications are: Cambridge Corporation, Aquila Pharmaceuticals, Antigenics, Q-VANT, Desert King, Croda International and Plant Bioscience. No applications were identified filed by Agenus, Phyton Biotech and Botanical Solutions.

## 4 Discussion

### 4.1 General overview of patenting trends for TB vaccine candidates

The present study aimed to develop the patent landscape for two TB vaccine candidates—MTBVAC and M72/AS01^E^—based on PCT applications, filed by institutions and companies involved in their development, and content analysis of their claims to characterize the scope of protection. The findings provide important insights on the potential access barriers regarding manufacturing, supply, and price, in case evidence generated in ongoing and future efficacy trials supports the regulatory approval of these vaccines.

As seen during the Covid-19 pandemic, the monopoly situation on medical countermeasures affected the R&D, production, supply and price (52). While patents on the technologies, such as vaccines, were one aspect allowing monopolistic situations (53, 54), other forms of IP—such as trade secrets—and access to know-how also emerged as key challenges to expand production through the diversification of manufacturers (55). Trends of international patent filing in Covid-19 vaccines—mRNA and viral vector platforms—have also shown a high number of applications, broad scope of protection, which covered not only different components of the vaccines but also future vaccine candidates, demonstrating the evergreening approach. Patent applications covering more components of the commercial vaccines were filed after the pandemic, showing a dynamic and prospective landscape, reinforcing the need for constant monitoring (56).

If filed and granted in countries, these patents could potentially be a barrier to the development of a technology platform, disincentivizing the manufacturer to produce affordable versions and potentially bringing legal risks for companies with similar technologies already approved or under development, even before the granting of a patent.

Importantly, the negotiations at the WHO for a Pandemic Agreement involved some of these issues, which are related to the findings in the present study, such as IPR; a system for pathogen access and benefits sharing; technology transfer; placing terms and conditions on research funding to ensure access at affordable prices to the fruits of scientific progress, especially when investments come from public sources (57). Addressing these IP barriers will require not only technical measures but also strong political will, global coordination, and advocacy to ensure that TB vaccines are treated as public goods.

Moreover, under the international human rights law, everyone holds the right to enjoy the benefits of scientific progress and its applications, which requires states to “make available and accessible to all persons, without discrimination, especially to the most vulnerable, all the best available applications of scientific progress necessary to enjoy the highest attainable standard of health” (58). The Committee on Economic, Social and Cultural Rights (59) has recognized that IP can negatively affect access to scientific benefits and applications (such as new medicines and vaccines) and distort the focus of R&D away from developing solutions for diseases like tuberculosis that disproportionately affect communities that are poor and disenfranchised. The Committee further highlighted the role of patent-backed monopolies in allowing innovators to set high prices that inhibit access and thereby prevent people from realizing the right to science. More recently, the Human Rights Council has called for interpreting international legal frameworks on IP against the obligations of states to ensure effective access to medicines, vaccines and other health products (60).

For MTBVAC, six PCT applications were identified (Figures 1, 2), of which there was a gap of 8 years between two applications (WO/2007/110462 and WO/2015/1444960). PCT application WO/2015/1444960 was filed in 2014 in Spain, coinciding with the beginning of the first clinical trial for this vaccine candidate, in 2013 (61). Half of these PCT applications were filed in selected countries (Table 1), such as Brazil, China, and India, which are not only high TB burden countries, but also have vaccine manufacturing capacity (62). The 20-year patent term for WO/2007/110462, which covers the API, is expected to expire in 2027. The other two applications covering pharmaceutical compositions (freeze-dried and aerosol) are expected to expire in 2039 and 2040, respectively.

During the Covid-19 pandemic, vaccine inequity between HIC and LMIC was a consequence of the monopoly situation over the vaccines from companies, which, by not transferring technology to countries with manufacturing capacity, have ensured that production fell short of global demand (63). In response, renewed attention has been given at the international level on the need to strengthen regional manufacturing capacity for vaccines in LMIC (64, 65). For the MTBVAC analysis, patents on the API (i.e., the genetically modified MTB strain) or pharmaceutical compositions in countries with existing manufacturing capacity are potential barriers to the production and supply of this vaccine candidate by alternative producers.

For M72/AS01^E^, 22 PCT applications filed by GSK were identified (Figures 4, 5), the majority of which is related to the adjuvant system. This patenting trend presents several access challenges if granted in countries. First, from a R&D perspective, patents covering the adjuvants may affect the development of other vaccines, either for TB or for other diseases, such as malaria and shingles (as the AS01 adjuvant system is part of two other licensed GSK vaccines, respectively, Mosquirix® and Shingrix®). Second, considering QS-21 is a natural resource, patents covering its process of isolation, purification or even semi-synthesis represent approaches of appropriating a supply found in nature, risking over time to be controlled by one company. Third, from an access perspective, patents on the key components of the vaccine as well as their production process may affect the opportunity of countries with existing vaccine manufacturing capacity to produce successful vaccines for regional supply, unless there is a willingness of the technology holder to license and transfer the technology to manufacturers in LMIC or for these countries to make full use of TRIPS flexibilities.

While patent applications for specific processes to obtain and purify adjuvants were filed in HIC, Brazil and India, those applications related to obtaining saponin were also filed in Chile and Mexico. This is probably related to the fact that the company responsible for supplying QS-21 (Desert King) from tree extraction is based in Chile and Mexico (66).

### 4.2 M72/AS01^E^ vaccine candidate: the development process and the role of GSK

GSK started the development of M72/AS01^E^ vaccine as an investigational candidate in the 2000s, and in 2012 signed an agreement with Aeras—a nonprofit product development partnership that received funding from the Bill & Melinda Gates Foundation and other private foundations as well as a wide range of governments—to jointly advance the clinical development of the vaccine through phase II (51). In 2018, Aeras and GSK published results of a phase IIb trial that showed M72/AS01^E^ conferred nearly 50% protection against developing bacteriologically confirmed pulmonary TB compared with placebo among 3,575 HIV-negative adolescents and adults previously infected by *M. tuberculosis* over 3 years of follow-up (67). This positive signal of efficacy revitalized TB vaccine development and led the WHO to call on stakeholders to rapidly undertake confirmatory evaluations in a phase III trial (68). Despite this successful phase IIb trial, GSK announced it would not advance M72/AS01^E^ to a phase III trial on its own (38) mainly due to profit (69–71), as this vaccine would be mainly sold to LMIC and non-profit organizations, making it less profitable than, for example, the shingles vaccine (Shingrix®) that could be sold in HIC, like the United States (71).

In 2018, Aeras transferred TB vaccine clinical research programs to IAVI (72), and, in 2020, GSK licensed rights to develop M72/AS01^E^ to the Gates Medical Research Institute (Gates MRI) but maintained rights to the AS01^E^ adjuvant (73, 74). GSK not only retained the control of the adjuvant, but also in the terms of the agreement, the company held the sales rights in wealthier countries (if the trials were successful). In this agreement, “GSK continues to provide the adjuvant component for clinical trials, and will also for the commercial product [if the phase III trial is successful]” (75) and is supposedly transferring the process to manufacture the antigen to Gates MRI.

Regarding the adjuvant, meanwhile GSK ensured the supply during the phase III trial (76), if the candidate gets approval, the demand will rise and the production would need to ramp up, securing enough for the other vaccines in the GSK portfolio presenting similar adjuvant systems (as Shingrix®). Furthermore, GSK unwillingness to share the “know how” for AS01^E^ production, due to the complexity of the process and for being a key component in many company vaccines (71), may raise serious questions in the TB community regarding the supply and prices in which the countries in need will be able to acquire the vaccine.

### 4.3 M72/AS01^E^ vaccine candidate: the role of the adjuvant and its components

MPL, one of the AS01 adjuvant system immune enhancers, was originally developed by Edgar Ribi in the 1970s, during his career at the Rocky Mountain Laboratories of National Institutes of Health’s National Institute of Allergy and Infectious Diseases and later at the company he founded, Ribi Immunochem (77). The company was acquired by Corixa Corporation in 1999 (78). In 2005, GSK acquired Corixa for US$ 300 million, controlling the production and supply of MPL (79, 80). Corixa Corp also played a key role in the development of the antigen M72 (which early in its development was called Mtb72F) (81).

The development of the adjuvant system started in the 1980s, when the US Army Medical Research and Development Command’s Walter Reed Army Institute of Research (WRAIR) was investigating a malaria vaccine. WRAIR brought in SmithKline Beechman (now GSK) to develop the vaccine candidate (82), whose clinical trials were then supported by the PATH Malaria Vaccine Initiative, with backing from the Gates Foundation. Liposomes containing MPL were developed in the WRAIR and gained interest for their potential as adjuvants (83). During the collaboration between GSK and WRAIR, various adjuvant combinations were investigated, with the combination of MPL and QS-21 emerging as the most promising (84). Carl Alving, a former investigator at the WRAIR, defined the time as “an exciting development,” until GSK filed patent applications covering the combinations at the EPO, essentially cementing ownership over the technology, and “The Army felt perhaps a little frustrated by that because we had introduced Glaxo to the field” (71).

QS-21, a purified fraction of *Quillaja saponaria* bark extract containing a mixture of two natural saponin isomers, was first identified and isolated in the late 1980s (48). The research took place in Cambridge Biotech Corporation that was later reorganized to form Aquila Biopharmaceuticals in the 1990s (85); which was acquired by Agenus (formerly Antigenics) in 2000 (86). No QS-21 PCT application filed by Agenus was found, but there is a filing by Antigenics in 2002 (Figure 6).

GSK got a license for QS-21 from Agenus (Stimulon®) for use in the AS01 adjuvant system in 2006, and in 2012 the two companies amended their licensing arrangement to give GSK additional rights to QS-21 (81). The deal included granting GSK the first right to negotiate to buy out Agenus or certain of its assets; Agenus got a non-refundable payment of US$ 9 million, as well as royalties (87). GSK gained control on QS-21 supply. In 2015, Agenus traded Stimulon® adjuvant rights to GSK for US$ 115 million to bolster its oncology portfolio. The conditions of the agreement included that GSK would pay off the debt taken by Agenus from Oberland Capital, with the royalties it would have owed Agenus under the previous arrangement. If GSK completed the payments, the rights would go back to Agenus, but if the adjuvant did not perform as expected, Agenus would pay the rest of the debt (88). In 2018, Agenus sold 100% of the royalties paid by GSK to HealthCare Royalty Partners (HCR) for US$ 190 million and US$ 40.35 million in milestone payments from HCR based on sales of GSK vaccines (89).

From 2019, GSK filed five PCT applications pursuing the protection of products and processes around QS-21 (Figure 4), ranging from “improved” extraction and purification methods to enzymatic modification of the saponins (e.g., conversion of QS-18 to QS-21) and methods to obtain QS-21 from plant cell culture. Most of these applications are also filed in Chile and Mexico, as an attempt to pursue control over the process to obtain QS-21.

The traditional method for producing QS-21 involved isolating the fraction from harvesting raw materials—the inner bark of *Quillaja saponaria* trees that are more than 10 years old (90). These trees grow in central Chile, but with the increasing demand, there is a growing concern about sustainable access to QS-21 (91) and the Chilean government has intervened with stricter laws. Desert King is based in Chile and is the leading company in the field, purchases the bark extract from small local growers and has invested in its own plantation (90, 91). This company was initially focused on producing and processing Yucca plant in Mexico.

As described in Figure 6, PCT applications from these companies related to saponins were identified since 1988. Desert King pursued PCT applications related to the process for obtaining saponins.

With an increasing global demand for the saponins, and their limited supply, the need for alternative and sustainable methods to produce QS-21 became glaring. Agenus subsidiary—SaponiQx—has focused on a plant cell culture-based manufacturing process, and, in 2019, got a grant of around US$ 1 million from the Gates Foundation for its development (86). The manufacturing process was developed using Phyton Biotech’s Plant Cell Fermentation (PCF®) Technology platform, under an exclusive partnership with Agenus, but no QS-21 PCT application filed by Agenus or Phyton Biotech was found. Stimulon® cultured plant cell (cpc) QS-21 produced in SaponiQx is now commercially available via InvivoGen, who markets the product (92, 93).

Besides efforts to improve the manufacturing process, additional players emerged to obtain QS-21. One of them is Botanical Solutions, a California-based startup that cultures *Quillaja saponaria* trees *in vitro* and has partnered with Croda Pharma to accelerate production of sustainable vaccine adjuvant QS-21 in 2023 (94). No QS-21 PCT application filed by Botanical Solutions was found, but there are three filings by Croda Pharma related to saponins in nanoparticles (Figure 6).

Q-VANT has also developed Q-SAP™ (Quillaja Sustainable Adjuvant Platform), a technology platform that enables varied Quillaja sourcing options and enhances production volumes (95). The two PCT applications from this company are from 2023 on vaccine compositions involving saponins and the process to obtain QS-21 from the entire biomass (Figure 6). In October 2024, Q-VANT has signed an agreement with In vitro Plant-tech AB, a Swedish plant cell cultivation company, whose technology platform and manufacturing facilities will permit the sustainable production of *Quillaja saponaria* biomass within bioreactors (96); and also with SPI Pharma, which includes an investment to expand Q-SAP™ technology, and a commercial agreement to “accelerate global adoption of their high-purity saponin adjuvants for veterinary and human vaccine formulations” (97).

Recently, researchers from John Innes Center have published the complete biosynthesis of QS-21 in a heterologous host (*Nicotiana benthamiana*) (98), and have partnered with Plant Bioscience Limited (PBL), who are leading commercialization of this project (99). PBL has filed several PCT applications about *Quillaja saponaria* biosynthesis (Figure 6).

Of note, Novavax has developed an adjuvant system called Matrix-M™ consisting of two different populations of physically stable nanoparticles mixed at a defined ratio (85% Matrix-A™ + 15% Matrix-C™). Matrix-A™ and Matrix-C™ contain different QS saponin fractions (100). The company obtains the saponins solely from Desert King (90). Therefore, GSK is no longer the only/main buyer of pharmaceutical-grade QS-21.

All the patenting trends by multiple applicants (Figures 4, 6) aiming to protect either the saponins, the processes involved in the isolation of some compounds (such as QS-21) or even their entire synthesis reflect an appropriation of a natural resource and an attempt to dispute the potential market of adjuvants such as AS01 system.

A possible future scenario is that as alternative methods for obtaining QS-21 advance and become the primary means of production, the supply would not be a challenge anymore, and the market dynamics could shift significantly. Companies may focus on synthetic QS-21 or similar derivatives, or fractions, with enhanced stability, efficacy, or bioavailability; and these variants might be eligible for patent protection as chemical compounds. The evolving landscape of saponin production and innovation could redefine the competitive space and IP strategies in this field.

## 5 Conclusion

The development of new TB vaccines is a promise that can change the course of this centenary epidemic. TB communities engaging in the development of these vaccine candidates will have a critical role to play in pursuing and addressing access issues throughout the entire development process to ensure that future vaccines will be timely and equitably available for those who need them in LMIC. This includes addressing IP barriers, including through the full use of public health TRIPS flexibilities, such as research exemption, a public health approach to patent examination, patent opposition and compulsory licenses.

In the context of the WHO Accelerator report (18), in which one of the key take-aways is on the need for public transparency, this study is part of this effort to improve information on IP on specific vaccines candidates, aiming to transform research outcomes into equitable access.

## Data Availability

The original contributions presented in the study are included in the article/Supplementary material, further inquiries can be directed to the corresponding author.
