# Supplementary material for: TB vaccine development: monitoring international patent filings to anticipate access challenges
Source: Front Public Health. 2026 Jan 16;13:1726153. doi: 10.3389/fpubh.2025.1726153 (PMC12855533; doi:10.3389/fpubh.2025.1726153)
Supplement: Supplementary file 1 [file Supplementary_file_1.docx]

Supplementary material:

Methodology: National and regional patent filings related to the previously included PCT applications were searched in CAS Patent Explorer database and in WIPO Patent Scope including all countries available. For a few cases, the status and national numbers were adjusted according to inputs from national search (India, Thailand, Ukraine and Indonesia). Whenever a patent application was not found in the databases used at the time of the search, it was not included in the tables below.

- **List of** **30 high tuberculosis burden countries (2024)**^^[[1]](#footnote-1)^^: Angola, Bangladesh, Brazil, Central Africa Republic, China, Congo, Democratic People’s Republic of Korea, Democratic Republic of the Congo, Ethiopia, Gabon, India, Indonesia, Kenya, Lesotho, Liberia, Mongolia, Mozambique, Myanmar, Namibia, Nigeria, Pakistan, Papua New Guinea, Philippines, Sierra Leone, South Africa, Thailand, Uganda, United Republic of Tanzania, Viet Nam, Zambia.
- **Regional patent offices considered in the search**: African Regional Intellectual Property Organization (ARIPO), African Intellectual Property Organization (OAPI), Patent Office of the Cooperation Council for the Arab States of the Gulf (GCCPO), Eurasian Patent Office (EAPO) and European Patent Office (EPO).

**Supplementary Table 1. National or regional patent applications on MTBVAC vaccine candidate (*Note: Countries in bold are among the 30 high TB burden countries).***

| **International publication number** | **Granted** | **Pending** | **Withdrawn/Abandoned** | **Rejected/Revoked** | **National filing without information about the status** |
| --- | --- | --- | --- | --- | --- |
| WO/2003/012075 | Spain (ES2316590 - expired, ES2192949 - expired)  United States of America (US7468190 - expired) | - | Austria (AT414761)  Australia (AU2002355780)  Canada (CA2456007)  Germany (DE60229962)  Japan (JP4153423)  Portugal (PT1428870)  EPO (EP1428870) | - | - |
| WO/2007/110462 | Austria (AT633719)  **Brazil (BRPI0709106)**  Canada (CA2647287)  **China (CN101405386)**  Germany (DE602007033030)  **India (IN268173)**  Japan (JP5324420)  Portugal (PT1997881)  Russia (RU2443773)  Spain (ES2433518)  United States of America (US8287886, US8642011)  EPO (EP1997881) | - | - | - | - |
| WO/2015/144960 | Spain (ES2549366) | - | - | - | - |
| WO/2018/006939 | - | - | - | - | - |
| WO/2019/158779 | Australia (AU2019221709)  **China (CN112449604)**  Germany (DE602019041418)  Japan (JP7388636)  Russia (RU2778094)  Spain (ES2967066)  United States of America (US11826411)  EPO (EP3755374) | Australia (AU2024203034)  **Brazil (BR112020016704)**  Canada (CA3091304)  **China (CN116751703)**  **India (IN202017038876, IN202318031011)**  Japan (JP2023153868)  United States of America (US20240382575)  EPO (EP4233912) | United States of America (US20230330201) | - | - |
| WO/2021/058831 | **China (CN114845732)** | Australia (AU2020355198)  Canada (CA3155558)  **India (IN202217022701)**  Japan (JP2022550722)  Mexico (MX2022003690)  Republic of Korea (KR1020220097398)  United States of America (US20230263837)  EPO (EP4340873) | EPO (EP3797791) | - | - |

Source: the authors, based on the patent status provided at WIPO Patent Scope and CAS Patent Explorer.

**Supplementary Table 2. National or regional patent applications on M72/AS01^E^ vaccine candidate filed by GSK. (*Note: Countries in bold are among the 30 high TB burden countries)***

| **International publication number** | **Granted** | **Pending** | **Withdrawn/ Abandoned** | **Rejected/ Revoked** | **National filing without information about the status** |
| --- | --- | --- | --- | --- | --- |
| WO/1994/000153 | Australia (AU661404 - expired)  Canada (CA2138997 - expired)  **China (CN1122530 - expired)**  Czech Republic (CZ282235 - expired)  Denmark (DK0671948 - expired)  Finland (FI946064 - expired, FI109767 - expired)  Germany (DE69313134 - expired, DE69327599 - expired)  Greece (GR3032742- expired, GR3025184 - expired)  Hong Kong (HK1010097 - expired, HK1022074 - expired)  Hungary (HU219808 - expired)  Israel (IL106109 - expired)  Japan (JP3755890 - expired)  Kazakhstan (KZ5698 - expired)  Malaysia (MY109278 - expired)  Morocco (MA22911 - expired)  Mexico (MX190367 - expired)  New Zealand (NZ253137 - expired)  Norway (NO317546 - expired)  Poland (PL170980 - expired)  Portugal (PT761231 - expired)  Republic of Korea (KR100278157 - expired)  Russia (RU02118164 - ceased)  Singapore (SG49909 - expired, SG90042 - expired)  Slovenia (SI9300335 - expired)  Slovakia (SK279188 - expired)  Spain ( ES2143716 - expired, ES2108278 - expired)  **South Africa (ZA1993/04504 - expired)**  Thailand (TH15255 - ceased)  Ukraine (UA40597 - expired)  United States of America (US5750110 - expired, US7147862 - expired)  EPO (EP0671948 - expired, EP0761231 - expired) | - | - | - | Austria (AT188613, AT156710)  Saudi Arabia (SA524)  **ARIPO (APAP 408)** |
| WO/1996/033739 | Algeria (DZ2026 - expired)  Australia (AU699213 - expired, AU693022 - expired)  Bulgaria (BG63491 - expired)  **Brazil (BRPI9608199 - expired, litigation)**  Canada (CA2217178 - expired  **China (CN1111071 - expired, CN1248737 - expired, CN1289065 - expired)**  Czech Republic (CZ296216 - expired)  Germany (DE69637254 - expired, DE69605296 - expired)  Denmark (DK0822831 - expired, DK0955059 - expired)  **Indonesia (IDP000005617 - expired)**  Japan (JP3901731 - expired)  Republic of Korea (KR100463372 - expired)  Malaysia (MY134811 - expired)  Morocco (MA23850 - expired)  Mexico (MX199930 - expired)  New Zealand (NZ305365 - expired)  Norway (NO322190 - expired)  Poland (PL184061 - expired)  Portugal (PT955059 - expired)  Romania (RO119068 - expired)  Saudi Arabia (SA475 - expired) Singapore (SG53407 - expired)  Slovakia (SK282017 - expired)  Slovenia (SI0955059 - expired, SI0822831 - expired)  Taiwan (TW515715 - expired)  **Thailand (TH27045 - ceased)**  Turkey (TR199701252 - expired)  Ukraine (UA56132 - expired)  **Vietnam (VN1068 - ceased)**  EAPO (EA000839 - expired)  EPO (EP0822831 - expired, re-examination, EP0955059 - expired)  **OAPI (OA10629 - expired)** | **India (IN2467/DEL/2007)** | - | EPO (EP0884056) | Argentina (AR001686)  Austria (AT186842, AT373487)  Cyprus (CY2588)  Greece (GR3031912)  Portugal (PT822831)  Spain (ES2140076, ES2293708)  United States of America (US20050214322, US6846489)  **ARIPO (APAP 771**) |
| WO/2003/028760 | EPO (EP1432442 - expired) | - | Australia (AU200233883)  Austria (AT350059)  Germany (DE60217374)  Spain (ES2279890)  United States of America (US20080292686) | Japan (JP2005507898) | Canada (CA2461924)  United Kingdom (GB0123580) |
| WO/2006/117240 | Algeria (DZP2007000744)  Australia (AU2006243357 - ceased)  **Indonesia (IDP000035400 - ceased)**  **India (IN296468)**  Mexico (MX297551, MX324839, MX326059)  Slovenia (SI1877426 - ceased, SI2426141 - ceased, SI2457926 - ceased)  **Vietnam (VN10015346)**  **South Africa (ZA200709209)**  EPO (EP1877426, EP2426141, EP2457926) | **Indonesia (ID201404894)** | Austria (AT543832, AT688625, AT689514)  **Brazil (BRPI0622304)**  Canada (CA2607715, CA2821389)  **China (CN101273055 - re-examination, CN106390108, CN105903008)**  Colombia (CO07114249A)  Germany (DE602006027434, DE602006043175, DE602006043238)  Denmark (DK1877426, DK2426141, DK2457926)  Estonia (EEE006763, EEE010044, EEE010045)  Spain (ES2381492, ES2524570, ES2524572)  Hong Kong (HK1116495)  Croacia (HRP20120331, HRP20141125, HRP20141184)  Israel (IL186654, IL215112)  Japan (JP5164830, JP5659207)  Republic of Korea (KR101352806, re-examination)  Norway (NO340206, NO340766, NO345071)  New Zealand (NZ562729)  **Philippines (PH12007502365, PH12013502449)**  Poland (PL1877426, PL2426141, PL2457926)  Portugal (PT1877426, PT2426141, PT2457926)  Singapore (SG136649)  Ukraine (UA98605)  United States of America (US10105430, US10350283, US10639361, US20170065696, US8470338, US9056913, US9655958)  EAPO (EA012576) | **China (CN102617739 - re-examination)**  Japan (JP2012065650, re-examination, JP2015057403)  Republic of Korea (KR102012008947, KR1020130110233 re-examination, KR1020150036658)  **Vietnam (VN20476)** | **Brazil (BRPI0611347)**  Cyprus (CY1112851)  Hungary (HUE013742)  Morocco (MA29678)  Slovakia (SKE11890, SKE17689) |
| WO/2007/068907 | Algeria (DZP2008000436)  Austria (AT632378, AT666084, ATE542543)  Australia (AU2006325377- ceased)  **Brazil (BRPI0619795)**  Canada (CA2633008, CA2633008)  Germany (DE602006027366, DE602006038535, DE602006039259, DE602006039390, DE602006040134, DE602006041549)  Denmark (DK1959992, DK2364720, DK2364724)  Estonia (EEE006449, EEE008665, EEE009449)  Croatia (HRP20120136, HRP20131057, HRP20140484)  **Indonesia (IDP000031710 - ceased)**  Japan (JP2009519309, JP5461015)  Republic of Korea (KR101363879, KR1020080079312)  Morocco (MA30023 - ceased)  Mexico (MX292604)  Malaysia (MY145943 - ceased)  **Philippines (PH12008501400)**  Poland (PL1959992, PL2364720, PL2364724)  Portugal (PT1959992, PT2364720, PT2364724)  Singapore (SG10201405533, SG10201405533, SG142872)  Slovenia (SI1959992, SI2364720, SI2364724)  Spain (ES2378471, ES2436645, ES2444623, ES2445170, ES2451573, ES2479165)  United States of America (US10039823, US10143745)  **Vietnam (VN10012047)**  EAPO (EA014353)  EPO (EP1959992, EP2364720, EP2364721, EP2364722, EP2364723, EP2364724)  GCCPO (GC0003025) | Costa Rica (CR10101) | Austria (AT640273, AT641179, AT649481)  **China (CN103405764)**  Hong Kong (HK1118477, HK1157219)  Israel (IL191703)  New Zealand (NZ568825, NZ596870)  Singapore (SG170127)  Taiwan (TWI457133)  Thailand (TH94291)  Ukraine (UA98612)  United States of America (US20080279926)  EAPO (EA018860) | Argentina (AR058543)  Colombia (CO08071881)  **China (CN102631670 - re-examination, CN103861100 - re-examination)**  **India (IN2608KOLNP2008)**  Japan (JP2013056927)  Norway (NO20082472)  Peru (PE0010982007) | Costa Rica (CR10118)  Cyprus (CY1112589, CY1114864, CY1115308)  Egypt (EG2008060966, EG2008061075, EG2008061074, EG2008061068)  Israel (IL229521)  Slovakia (SKE15369) |
| WO/2010/142685 | Austria (AT674188)  Australia (AU2010257538)  **Brazil (BRPI1012890)**  Canada (CA2764421)  **China (CN102458457 - re-examination, CN104367997)**  Germany (DE602010016998)  Denmark (DK2440242)  Estonia (EEE009672),  Croatia (HRP20140792)  Israel (IL216231)  **India (IN279396)**  **Indonesia (IDP000040492 - ceased)**  Japan (JP5647677, JP5977789, JP6261647)  Republic of Korea (KR101473686)  Morocco (MA33412 - ceased)  Mexico (MX325138)  Malaysia (MY159819 - ceased)  Norway (NO2440242)  Poland (PL2440242)  Portugal (PT2440242)  Serbia (RS53550)  Singapore (SG176638)  Slovenia (SI2440242)  Spain (ES2494442)  San Marino (SMT201400134)  **South Africa (**[**ZA201108507**](https://patentexplorer.cas.org/patent_view/view?patentId=30733e12-ed4d-47cb-a265-92309d87d495&related_id=7071f813-b279-41dd-950e-eb83ce99dfc4&_type=family&_familyType=simple&page=4&rows=20&country=AT%2CAU%2CBR%2CCA%2CCL%2CCN%2CCO%2CCY%2CDE%2CDK%2CDO%2CEA%2CEE%2CEP%2CES%2CGB%2CHK%2CHR%2CHU%2CIL%2CIN%2CJP%2CKR%2CMA%2CMX%2CMY%2CNO%2CNZ%2CPE%2CPL%2CPT%2CRS%2CSG%2CSI%2CSK%2CSM%2CTH%2CUA%2CUS%2CVN%2CWO%2CZA)**)**  EAPO (EA024074)  EPO (EP2440242) | Chile (2011003113)  Costa Rica (CR2011000661) | Colombia (CO6440528)  Hong Kong (HK1162974)  New Zealand (NZ596503)  **Thailand (TH125812)**  Ukraine (UA104888)  United States of America (US20180021417,  US20120087976) | Peru (PE0003192012)  **Vietnam (VN30393)** | Algeria (DZP2011000890)  Cyprus (CY1115522)  Dominican Republic (DOP2011000346)  United Kingdom (GB0910046)  Hungary (HUE021538)  Slovakia (SKE17056) |
| WO/2011/144645 | Austria (AT1153491)  **Brazil (BR112012028930)**  Canada (CA2800368)  **China (CN102933606)**  Germany (DE602011060335)  Denmark (DK2571902)  Croatia (HRP20191685)  Hungary ( HUE047042)  **India (IN322114)**  Japan (JP6271619)  Lithuania (LT2571902)  Norway (NO2571902)  Poland (PL2571902)  Portugal (PT2571902)  Slovenia (SI2571902)  Spain (ES2748512)  United States of America (US9499639)  EPO (EP2571902) | - | United States of America (US20130066064) | Japan (JP2013527289) | Cyprus (CY1122071)  United Kingdom (GB201008401)  Slovakia (SKE32218) |
| WO/2012/080370 | Austria (AT1160227, AT774315, AT784610)  Australia (AU2011343368 - ceased)  **China (CN103249431, CN103260642)**  Germany (DE602011023279, DE602011024702, DE602011060976)  Denmark (DK2651436, DK2651437,  DK3023106)  Estonia (EEE011777, EEE012020, EEE018244)  Hungary (HUE027932, HUE028452)  **Indonesia (IDP000040568 - ceased)**  Japan (JP5951634)  Montenegro (ME02380)  Mexico (MX344280)  Malaysia (MY161412 - ceased)  Norway (NO2651436, NO2651437, NO3023106)  Poland (PL2651436, PL2651437, PL3023106)  Serbia (P-2016/0225)  Singapore (SG190937)  Slovenia (SI2651436, SI2651437, SI3023106)  San Marino (SMT201600118)  Spain (ES2567190, ES2574403, ES2748651)  United States of America (US10441648, US9352030)  **South Africa (ZA201304014, ZA201304015)**  EAPO (EA027504)  EPO (EP2651436) | - | Argentina (AR084285)  **Brazil (BR112013014598)**  **China (CN106822882, CN106822883)**  Hong Kong (HK1190618, HK1190619)  Croatia (HRP20160362, HRP20160606, HRP20191843)  **Indonesia (ID201402335)**  **India (IN342832)**  Israel (IL226450)  Lithuania (LT3023106)  **Philippines (PH12013501204)**  Portugal (PT2651436, PT3023106)  Republic of Korea (KR1020140029376)  Serbia (RS54687)  Singapore (SG190731)  **Thailand (TH143305)**  Taiwan (TW201305192)  Ukraine (UA110806)  United States of America (US20160220656, US20190183998)  EPO (EP3593813) | Colombia (CO6751287)  **Vietnam (VN35845, VN36110)** | Canada (CA2819298)  Cyprus (CY1122187)  Hungary (HUE045766)  Malaysia (MYPI2013700990)  Slovakia (SKE20945, SKE21285, SKE32386)  Uruguay (UY33802) |
| WO/2012/080369 | Austria (AT1160227, AT774315, AT784610)  Australia (AU2011343367 - ceased)  **China (CN103249431, CN103260642)**  Germany (DE602011023279, DE602011024702, DE602011060976)  Denmark (DK2651436, DK2651437,  DK3023106)  Estonia (EEE011777, EEE012020, EEE018244)  Hungary (HUE027932, HUE028452)  **Indonesia (IDP000040568 - ceased)**  Israel (IL226448)  Japan (JP5951633)  Republic of Korea (KR101951894 - re-examination)  Montenegro (ME02380)  Mexico (MX344706)  Malaysia (MY161412 - ceased)  Norway (NO2651436, NO2651437, NO3023106)  Poland (PL2651436, PL2651437, PL3023106)  Singapore (SG190937)  Slovenia (SI2651436, SI2651437,\| SI3023106)  San Marino (SMT201600118)  Spain (ES2567190, ES2574403, ES2748651)  United States of America (US10441648, US9730992)  **South Africa (ZA201304014, ZA201304015)**  EAPO (EA027920)  EPO (EP2651437, EP3023106) | - | Argentina (AR084285)  **China (CN106822882, CN106822883)**  Hong Kong (HK1190618, HK1190619)  Croatia (HRP20160362, HRP20160606, HRP20191843)  **Indonesia (ID201402335)**  Lithuania (LT3023106)  **Philippines (PH12013501203)**  Portugal (PT2651436, PT3023106)  Serbia (RS54687)  Singapore (SG190731)  **Thailand (TH143304)**  Taiwan (TW201305192)  Ukraine (UA110118)  United States of America (US20160220656, US20190183998)  EPO (EP3593813) | **Brazil (BR112013014599)**  Colombia (CO6751288)  **India (IN1651KOLNP2013)**  Republic of Korea (KR1020190022897)  **Vietnam (VN35845, VN36110)** | Canada (CA2819297)  Cyprus (CY1122187)  Hungary (HUE045766)  Israel (IL226450)  Malaysia (MYPI2013700990)  Slovakia (SKE20945, SKE21285, SKE32386)  Uruguay (UY33802) |
| WO/2015/150567 | Australia (AU2015239025)  Canada (CA2943007)  **China (CN106456738)**  Germany (DE602015062843, DE602015086326)  Israel (IL247493)  Italy (IT202100015722)  Japan (JP6645982,  JP6655549)  Republic of Korea (KR102762440)  Mexico (MX401336)  Singapore (SG11201607086)  United States of America (US10624961, US10688168, US11951161)  Spain (ES2853773, ES2961840)  **South Africa (ZA201605955)**  EAPO (EA037405)  EPO (EP3125929, EP3125930) | - | Argentina (AR099960)  Austria (AT1340160)  Belgium (BE1022355)  **Brazil (BR112016022463)** | **China (CN106456739)**  **India (IN201617031463)**  Republic of Korea (KR1020160132115 - re-examination) | **Brazil (BR112016022787)**  Canada (CA2943711)  United Kingdom (GB201405921)  **India (IN201617030399)**  Mexico (MX2016012932,  MX2022013912)  Malaysia (MYPI2015703754) |
| WO/2017/102737 | Belgium (BE1024188)  Germany (DE602016092681)  United States of America (US10702594, US11304999)  EPO (EP3389698) | - | - | - | United Kingdom (GB201522068) |
| WO/2018/104313 | Belgium (BE1025160)  **China (CN110035770)**  Germany (DE602017083938)  Japan (JP7136777)  Spain (ES2988600)  United States of America (US10695424)  EPO (EP3551222) | **Brazil (BR112019011286)**  Canada (CA3045952) | **India (IN544265)** | - | Mexico (MX2019006728) |
| WO/2018/114892 | United States of America (US10881731, US11679153) | EPO  (EP3558357) | Belgium (BE1025119)  **China (CN110290806)** | - | United Kingdom (GB201621686) |
| WO/2018/219521 | United Kingdom (GB2577232,  GB2600652, GB2600653, GB2600654)  Ireland (IE20190086, IE87413, IE87414, IE87439)  Japan (JP7291633),  United States of America (US12016919) | Canada (CA3063954)  **China (CN111032080)**  Ireland (IE20190084)  Mexico (MX2023012979, MX2023012980,  MX2023013020),  United States of America (US20240293538)  EPO (EP3630176) | Germany (DE112018002827)  Japan (JP2023110040)  **India (IN201917053739)** | France (FR3066920) | **Brazil (BR112019025193)**  Mexico (MX2019014319) |
| WO/2018/206776 | United States of America (US10973900),  **South Africa (ZA201907459)** | **India (IN201917049171**)  EPO (EP3624835) | - | **China (CN110891595)** | United Kingdom (GB201707700) |
| WO/2019/106191 | United States of America (US11744890, US12138307) | **Brazil (BR112020010635)**  Canada (CA3083059)  Chile (CL202001439)  Hong Kong (HK40033428)  Mexico (MX2020005480)  EPO (EP3717005) | **China (CN111372604)**  Japan (JP2023182592)  United States of America (US20240024467)  **India (IN202017023446)** | Japan (JP2021504429 - re-examination) | **-** |
| WO/2019/106192 | Mexico (MX413557)  United States of America (US11591364, US12227540) | **Brazil (BR112020010790)** Canada (CA3083078)  Chile (CL202001440)  **China (CN111670044)**  Hong Kong (HK40034658)  **India (IN202017023959)**  EPO (EP3717001) | - | Japan (JP2021504424 - re-examination,  JP2024001012) | - |
| WO/2021/224205 | - | Canada (CA3181627)  Hong Kong (HK40089408)  **India (IN202217059500)** Mexico (MX2022013855)  United States of America (US20230277657)  EPO (EP4146378) | **China (CN115485057)**  Japan (JP2023524136) | - | **Brazil (BR112022020660)** |
| WO/2022/122830 | - | **Brazil (BR112023010982)** Canada (CA3203278)  Hong Kong (HK40095922)  **India (IN202317037525)** Japan (JP2023553904)  Mexico (MX2023006769),  United States of America (US20240026407)  EPO (EP4259814) | - | - | Chile (CL202301630) |
| WO/2023/020994 | - | - | - | - | - |
| WO/2023/066885 | - | Canada (CA3233086)  Chile (CL202401193)  **China (CN118215502)**  Hong Kong (HK40109671)  **India (IN202417036649)**  Japan (JP2024538802)  EPO (EP4419141) | - | - | United Kingdom (GB2022008339) |
| WO/2023/242187 | - | Chile (CL202403833) **China (CN119403935**) Hong Kong (HK40119226)  **India (IN202417095096)** United States of America (US20250163486)  EPO (EP4540401) | - | - | Canada (CA3253348) |

Source: the authors, based on patent status analysis from WIPO Patent Scope, CAS Patent Explorer, and national searches (India, Thailand, Ukraine, Indonesia).

1. <https://www.who.int/teams/global-programme-on-tuberculosis-and-lung-health/tb-reports/global-tuberculosis-report-2024/tb-disease-burden/1-1-tb-incidence#fig--1-1-11> [↑](#footnote-ref-1)
